# Hedging Medical Spending Growth: An Adaptive Expectations Approach

**DOI:** 10.11114/afa.v2i2.1595

**Published:** 2016-05-06

**Authors:** Robert D. Lieberthal

**Affiliations:** 1 Jefferson College of Population Health, Thomas Jefferson University, Philadelphia, PA; 2 Sidney Kimmel Cancer Center, Thomas Jefferson University, Philadelphia, PA

**Keywords:** Medical spending growth, Cost curve, Healthcare finance, Long-term health insurance

## Abstract

Long-term health insurance provides consumers with protection against persistent, negative health shocks. While the stochastic rise in medical spending growth may make some health risks harder to insure, financial assets could act as a hedge for medical spending growth risk. The purpose of this research was to determine whether such hedges exist. The results of this study were two-fold. First, the asset classes with the strongest statistical evidence as hedges were bonds, not stocks. Second, any strategy to hedge medical spending growth involved shorting assets i.e. betting against the bond or stock market. Health insurers writing long-term contracts should combine the use of hedges in the bond market with of portfolio diversification, and may benefit from health policies to moderate the uncertainty of medical spending growth.

## 1. Introduction

### 1.1 Background

Medical spending growth, also known as medical trend or the healthcare cost curve, describes the phenomenon whereby medical spending rises annually. This growth rate is an important economic feature of the macroeconomics of developing countries, because healthcare is such a large share of these economies and is growing faster than the economy. It is also an important consideration for public policy, both because of the increasing share of government budgets devoted to healthcare and because of the need for private insurers and consumers to finance rising spending.

### 1.2 Importance of the Problem

The long-term increase in medical spending features a large degree of variation in medical spending growth. The growth rate is stochastic, varying substantially over time. In the U.S., for example, a number of years of low growth in spending growth during the 1990’s were followed by a number of years with high growth rates during the 2000’s. Year-to-year, this growth rate varies substantially, which has been extensively documented and researched. Government economists and actuaries make forecasts in order to set short, medium, and long-term budgets. Prior examples include estimates produced by the CBO for their budgeting purposes (Congressional Budget Office, 2010) and the Society of Actuaries technique for modeling long run healthcare cost trends (Getzen, 2007). There are also similar methodologies used in other developed countries (Astolfi, Lorenzoni & Oderkirk, 2012). Prior studies focused on forecasting average long-term rates of growth, and the determinant of growth rates. Variation around the trend line was generally expressed as a forecast error.

### 1.3 Related Literature

A small number of studies have also addressed the issue of how to hedge the risk generated by stochastic medical spending growth. Jennings et al. ask whether healthcare assets hedge healthcare liabilities, and conclude that they do not (Jennings, Fraser, & Payne, 2009). Markets for providing a direct hedge through futures contracts have been set up and studied, but have not succeeded (Cox & Schwebach, 1992). The question of whether these variations can be hedged by any traded financial asset by searching over the wider universe of traded assets (securities), has not been addressed in the literature.

Stochastic growth in medical spending is a major risk for consumers, health insurance companies, and governments. This risk is detrimental to consumers because it raises the cost of insurance and hampers consumer attempts to budget for the future. Health insurance companies that offer protection against this risk for consumers are responsible for pricing and managing this risk. Long-term care insurance, guaranteed renewable health insurance, and workers compensation insurance are three forms of insurance that price and manage this risk over the long-term i.e. more than one year (Herring & Pauly, 2006; Cutler, 1993; Feldblum, 1993). Thus, it would valuable to health insurers to find and utilize such hedges if they exist. If they do not exist, certain health risks could be uninsurable. As a result, the availability of hedges is a concern for public policy, as governments may decide to foster such hedges or to take on long-term health insurance through social insurance programs.

### 1.4 Hypotheses and Their Correspondence to Research Design

The purpose of this study was to determine whether variation in medical spending growth could be hedged with traded stocks or bonds. The goal was motivated by the investment problem that an insurance company that must pay future claims for medical care. The settings of long-term care, guaranteed renewability, and workers compensation highlighted are generalizable to any situation where an insurer makes a commitment to pay for future care based on the future cost of care. Such a commitment generates a long-term liability linked to the level of medical spending, and thus is sensitive to medical spending growth.

The main research questions this analysis considered were threefold. First, do hedges for medical spending growth exist? In other words, were there financial assets whose returns fluctuated with the stochastic portion of medical spending growth? The second question was, what would the hedging assets be? Were specific asset classes, such as healthcare stocks, good hedges? Was there any asset that is a hedge? Third, does the hedge involve buying such assets, i.e. ‘going long’, or selling short such assets?

In order to answer these questions, this analysis proceeded with the following steps. First, a model was selected to determine the unpredictable portion of medical spending growth. This analysis utilized an adaptive expectations model that allowed for a range of errors so that the results would not be overly reliant on a single specification. Then, this model was applied to health insurance spending data to generate the unexpected portion of spending growth. Data for a large number of broad asset classes was gathered, and used to calculate returns in excess of the medical spending growth rate. Excess returns were used to test each class of assets for its potential as a hedge. Positive coefficients for healthcare securities were hypothesized based on the conjecture that there would be a positive correlation between above trend spending growth and returns to healthcare securities. Negative correlations with the overall market and bonds were hypothesized based on the results of Bodie, 1976 that the way to hedge general inflation is to short common stocks.

## 2. Method

### 2.1 Model

The position of a risk neutral health insurance company was assumed in order to focus the attention on the investment problem. The health insurer is a passive ‘pass through’ entity that offers an actuarially fair long-term health insurance contract, and thus takes on the risk associated with volatility in medical spending growth. That price does not include an economic adjustment for the price of the risk in medical spending growth, since the insurer is assumed to have sufficient capital, or access to capital, in order to have reserves to pay for unexpectedly large claims. These assumptions focused the investigation on the problem that motivated this analysis, which is the investment problem related to hedging medical spending growth. Other considerations, such as the possibility of pricing in economic risk and active attempts by the insurer to shift risk back onto the consumer or onto other entities are important but beyond the scope of this analysis.

Next, an adaptive expectations model was chosen as the data generating function for medical spending growth. In the model chosen, the rate of medical spending growth is a long-term average, or ‘trend’, that the insurer forecasts before writing health insurance. The experienced rate of medical spending growth fluctuates around this rate. Considering the more recent period of 1982–2008 tend rate, medical spending growth is 6–8% depending on the benefits included, with significant fluctuation around the long-term average rate (see Table 1). An insurer that assumed 7% growth over this period would have been right on average, and would have experienced several years of above and below average growth rates.

Financial returns are assumed to include all available information (Malkiel & Fama, 1970). Crucially, information on the history of medical spending and medical spending growth, and the expectation of future growth rate, is assumed to be fully priced into current prices for stocks and bonds. In this study, excess returns on assets are used as the dependent variable of interest. The insurer is not interested in absolute returns, but rather returns relative to the benchmark of medical spending growth. The insurer would willingly give up higher financial returns on investments (reserves) in years when spending growth is below trend in order to obtain higher return when spending growth is above trend.

### 2.2 Estimation

In this study, an adaptive expectations model was used to determine deviations from trend. The long-term rate of growth is equal to the long-term rate of growth from the prior period, adjusted to reflect the difference between the long-term rate of growth and the experienced rate of growth in the prior period. The updating equation is given by equation 1:

Equation 1 Adaptive expectations updating equation

(1) $$\bar{D}(t)=\bar{D}(t-1)+\theta (D(t-1)-\bar{D}(t-1))$$

where

*D̄*(*t*) is the long term rate of growth at time *t*
*D*(*t*) is the experienced rate of growth at time *t*

This specification was originally proposed by Bodie, 1976, to determine whether common stocks hedge price inflation. The updating factor θ for the adjustment ranges from 0 to 1. The choice of updating factor θ determines the time series deviations from trend in the model. θ is not known a priori. Estimates of *D̄*(*t*) , the expected rate of growth, are calculated over a range of values for θ. *d*(*t*)=*D*(*t*)−*D̄*(*t*) are the unanticipated shocks that result, and differ by choice of θ. Therefore, this model generates a range of shocks for a range of possible updating factors.

Next, the long-tem rate of growth in each period was used to determine the excess rate of return on assets (see Equation Equation 1). The return on assets was not fit to a model in order to generate unanticipated returns, reflecting the efficient market model for asset returns. Excess returns reflected the objective function of the insurer to generate returns in excess of the long-term trend rate in order to pay for higher than expected claims from returns on investments. Absolute returns were used as an alternative specification that reflected the fact that investors actually receive absolute, not excess returns.

Equation 2 Excess returns on assets

(2) $$R_{e}(t)=R(t)-\bar{D}(t)$$

The relationship between excess returns and the deviations from the trend was assessed to determine which assets hedged spending growth. If the regression coefficient α_1_ in Equation 3 is significant, then the return index used to calculate excess returns is a good hedge. The sign of the coefficient indicates whether the hedging position is long or short.

Equation 3 Regression of excess returns on deviations

(3) $$R_{e}(t)=\alpha_{0}+\alpha_{1}d(t)$$

### 2.3 Data

The Centers for Medicare and Medicaid Services (CMS) provides the National Health Expenditure Data, which tabulates medical spending per insurance plan enrollee. The data includes per capital spending by private plans and Medicare, and further splits the data into all benefits and common benefits provided by both plans (for example, Medicare did not offer drug benefits until 2003 (Centers for Medicare and Medicaid Services, 2011)). Medical spending growth was defined for this study in terms of spending on private insurance plan enrollees. 1982 was chosen as the starting year based on when the prospective payment system (PPS) was implemented by Medicare, as well as based on graphical analysis. Private insurance growth rates are substantial with a high degree of variance (see Table 1). There was also not a single year of negative spending growth in the data. The data allowed evaluation of hedges for the perspective of this study, health insurers. Spending rose due to changes in price and quantity of medical care delivered, the composition of which is reflected in the insured spending time series.

Asset return data came from two sources. Statistics on bond returns are shown in Table 2. Fama-French return factors were used to determine the risk-free rate. The risk-free rate was based on one-month Treasury bills (Fama & French, 1993; Fama & French, 2010). Bond return data on total returns for ten-year government bonds and Moody’s index of AAA rated corporate bonds came from the Global Financial Data Total Return database (Global Financial Data, 2011).

Initial statistics on stock returns are shown in Table 3. Fama-French factors were used to generate returns for the stock market, healthcare stocks as a whole, and healthcare subsectors stock returns. The Fama-French returns are value weighted and cover stocks on the major U.S. exchanges (Fama & French, 2010). Health sector returns have higher mean returns than the market but also higher variance.

## 3. Adaptive Expectations Results

The results for the adaptive expectations model are shown in Table 4. The model generated long-term trends that are in line with experience over the time horizon used. Using data from 1982–2008, the long-term expected trend for spending growth was between 8.35–10.53%. Using a larger updating factor, meaning adapting expectations to more current data more quickly, generated the lower expectations of long-term medical spending growth, reflecting the fact that medical spending growth rates have been lower more recently.

There was a substantial amount of variation around the trend line. As shown in Table 5, the errors were negative, which reflects the slowing of trend over the time period used. The standard deviation of errors is the measure of the degree of variation around the trend line, which is the risk the insurance company wants to address through hedging. The standard deviation across updating factors were in a tight range from 2.97–3.21%. Thus, the results of the model on the riskiness of medical spending growth were not dependent on the choice of θ.

Table 6 contains the summarized results from regressing the errors against excess returns as shown in Equation 3. None of the stock market assets was a statistically significant hedge for stochastic medical spending growth across all values of θ. That includes the healthcare stock returns. For the broad market, the regression coefficients were strongly negative across all values of θ, as was true for health and all health subsectors with the exception of health services (health services included hospitals, doctors’ offices, and other healthcare facilities). Thus, if equity assets are good hedges, then they should be sold short in order to hedge the risk of unexpected growth in medical spending. This result is consistent with the canonical results of Bodie, 1976, and Fama & Schwert, 1977.

Table 7 contains the results for bonds from regressing the errors against excess returns as shown in Equation 3. The results for corporate bonds and the risk-free rate are both statistically significant and negative. Since the AAA corporate bond series represented total returns, the statistically significant results and size of the coefficient suggests shorting high quality corporate bonds as a way to hedge against medical spending growth. The results for government bonds are marginally significant for some values of θ, indicating this asset class may have been a less efficient hedge. The negative coefficient indicates that hedging strategies including these bonds should also utilize short sales. The strong, statistically significant coefficients on the risk-free rate mean that these instruments may be a partial hedge, because short-term government bonds are more likely to have a lower yield when medical spending growth is above trend. Thus, a leveraged investment strategy that shorts these bonds may provide a hedge against medical spending growth.

Additional alternative specifications of absolute returns were generated, with similar results except for the corporate bond and risk-free rates. Using absolute rather than excess returns does not alter the findings for stocks, both in terms of statistical significance and coefficients. The results do become statistically insignificant for the risk-free rate and corporate bonds. Thus, those results are more sensitive to the specification used.

Limitations considered included a small sample size and possible trend breaks over time. The sample size is the nature of financial and macroeconomic time series data, which requires many years of annual data in order to generate statistically significant results. Another possibility is that there are additional trend breaks in the time series within the 1982–2008 window. This could also account for the lack of statistical significance in the results. Additional specifications, which were not tested in this analysis, would involve additional asset classes and using assets in combination.

## 4. Discussion and conclusions

The main finding of this study is that returns to bonds are inversely related to unexpected changes in medical spending growth rates. This finding suggests important implications for insurance practice as discussed below. It also implies a negative finding about the use of equities as a hedge for changes in medical spending. In particular, it appears that returns to healthcare stocks, such as those for health insurance companies, hospitals, biopharmaceutical companies, and other publicly traded healthcare companies are not correlated with unanticipated changes in medical spending. Medical spending is also highly variable in this study, which is concordant with results of other studies into the “cost curve” for medical spending.

The main implication of the results is that insurance companies should use a hedging strategy of shorting corporate bonds to protect against stochastic medical spending growth. Insurance companies may want to combine this strategy with an overall diversified investment strategy to manage long-term health insurance contracts. Prudent investing would include the use of ample reserves, consistent with the theory of optimal reserves (Munch & Smallwood, 1981). The adaptive expectations model identified a sizeable risk, and the insurance company’s investment policy options to reduce the risk are limited. Health insurance companies should be conservative about which long-term risks it takes on. This includes both health insurance policies, like guaranteed renewable and long-term care insurance, and casualty lines with a health liability, such as workers’ compensation insurance and health reinsurance.

The limitations of hedging mean that the properties of medical spending growth determine which risks are insurable. In other words, public policy that makes spending growth less volatile will enhance the ability of insurance companies to write longer-term health insurance policies. It will also enhance public insurance programs; while government budgets for many programs are set on an annual basis, longer-term budgeting is hampered by unanticipated changes in medical spending.

In the U.S. context, it is important to consider whether the Patient Protection and Affordable Care Act (PPACA), will result in more ‘long-term’ insurance. If the law can reduce ‘churning’ by individuals from insured to uninsured status, or between health insurance companies, then it could lead to implicitly longer-term arrangements between individuals and their health insurance company. That could make medical spending growth risk more important, as insurers start to provide long-term health insurance by default. Hedging medical spending growth may then become more important to health insurers. That would be true even if the PPACA ‘bends the cost curve’, as there could still be significant volatility around a lower trend growth in medical spending.

Insurers would be well served to adequately reserve for future costs, which is the essence of the theory of optimal reserves. However, that theory assumes that proper investment opportunities for reserves exist. The implication of this study is that the best investment opportunity for reserves is in diversified, low cost investments that focus on retaining the capital value of reserves. Active management should concentrate on short sale opportunities, so that if unanticipated jumps in medical spending occur, insurers may be able to manage their higher claims costs with excess returns generated by the hedging strategy identified in this analysis. Future research should focus on the possibility that the size of the risk, and limited number of hedges, could make some risks uninsurable. The place of public policy could then be to make such risks insurable, either by providing hedging assets or taking such risks on directly.

## Figures and Tables

**Table 1:** Rate of change in per capita nominal insurer expenditures

	All benefits		Common benefits	

Insurance type	Medicare	Private	Medicare	Private
Mean	7.08	7.91	6.08	7.33
Standard deviation	3.50	3.56	2.83	3.22
Skewness	0.07	0.35	0.59	0.16
Kurtosis	3.76	2.22	4.19	2.09
Minimum	−1.50	1.87	0.08	2.18
Maximum	15.21	15.33	14.1	13.69
25th percentile	4.90	4.89	4.38	4.84
75th percentile	8.96	10.39	7.37	9.62

Descriptive statistics for the rate of change in insured healthcare expenditures on a percentage basis from 1982–2008. “All benefits” includes all spending, while “common benefits” includes only that spending covered by both Medicare and private health insurance plans.

**Table 2:** Bond monthly nominal returns

	Bond class		

Returns	10-Year Government Bonds	AAA Corporate Bonds	Risk-Free Rate
Mean	0.84	0.88	0.42
Standard deviation	2.42	1.91	0.21
Skewness	0.22	0.52	0.43
Kurtosis	3.75	5.54	3.22
Minimum	−6.94	−4.73	0.02
Maximum	8.64	8.55	1.13
25^th^ percentile	−0.65	−0.13	0.28
75^th^ percentile	2.35	1.87	0.54

This table shows the summary statistics for returns to three classes of bonds from 1982–2008: long-term government bonds, highly rated corporate bonds, and short term government bills used to calculate the risk-free rate. Returns were calculated on a continuous log basis and are expressed in percentage terms.

**Table 3:** Stock monthly nominal returns

	Equity class				

Returns	Market	Health	Health services	Medical equipment	Drugs
Mean	0.91	1.12	0.83	1.05	1.21
Standard	4.48	4.83	6.92	5.29	5.01
deviation					
Skewness	−0.91	−0.19	−0.36	−0.50	−0.10
Kurtosis	6.21	4.24	4.75	4.85	3.84
Minimum	−22.54	−20.47	−31.50	−20.56	−19.10
Maximum	12.85	16.54	20.49	16.31	16.37
25^th^ percentile	−1.70	−2.03	−3.51	−1.82	−1.89
75^th^ percentile	3.91	4.00	5.11	4.46	4.38

This table shows the summary statistics for returns to four classes of equities from 1982–2008: the broad market, a basket of healthcare securities, and three subsectors of the healthcare market, services, equipment, and drugs. Returns were calculated on a continuous log basis and are expressed in percentage terms.

**Table 4:** Adaptive expectations average rates for per capita nominal insurer expenditures

	Updating factor (θ)									

	0.1	0.2	0.3	0.4	0.5	0.6	0.7	0.8	0.9	1.0
Mean	10.53	9.47	9.03	8.80	8.65	8.55	8.48	8.42	8.38	8.35
Standard deviation	2.19	2.57	2.72	2.85	3.01	3.16	3.32	3.46	3.60	3.73
Skewness	0.22	0.42	0.42	0.36	0.33	0.32	0.33	0.34	0.34	0.33
Kurtosis	1.59	1.86	1.97	1.89	1.80	1.76	1.79	1.88	2.02	2.19
Minimum	7.52	6.32	5.49	4.98	4.64	4.47	4.38	3.50	2.66	1.87
Maximum	14.19	14.35	14.37	14.34	14.35	14.44	14.62	14.86	15.16	15.49
25^th^ percentile	8.40	7.19	6.74	6.33	6.05	5.46	4.99	5.07	5.06	4.98
75^th^ percentile	12.22	11.61	11.71	11.21	11.28	11.63	11.29	10.96	10.66	10.57

This table shows the summary statistics for expected insurer expenditures for the time period 1982–2008. The expected rates vary based on the updating factor θ, and the table shows the rates for values of θ from 0.1 to 1.0.

**Table 5:** Adaptive expectations errors for per capita nominal insurer expenditures

	Updating factor (θ)									

	0.1	0.2	0.3	0.4	0.5	0.6	0.7	0.8	0.9	1.0
Mean	−2.62	−1.57	−1.13	−0.89	−0.74	−0.64	−0.57	−0.52	−0.48	−0.44
Standard deviation	3.21	3.29	3.27	3.22	3.16	3.10	3.05	3.01	2.99	2.97
Skewness	−0.23	−0.13	0.02	0.14	0.22	0.28	0.33	0.36	0.38	0.41
Kurtosis	3.65	3.91	4.10	4.41	4.75	5.01	5.10	4.99	4.76	4.48
Minimum	−11.27	−10.51	−9.86	−9.34	−8.96	−8.66	−8.41	−8.19	−7.97	−7.73
Maximum	3.70	5.48	6.76	7.60	8.10	8.33	8.33	8.13	7.76	7.21
25^th^ percentile	−3.94	−3.23	−2.64	−2.69	−2.45	−2.32	−2.27	−2.01	−2.31	−2.46
75^th^ percentile	−0.56	0.81	0.71	0.89	1.03	1.04	0.99	0.92	0.96	0.99

This table shows the summary statistics for errors based expected insurer expenditures for the time period 1982–2008. The error rates vary based on the updating factor θ, and the table shows the rates for values of θ from 0.1 to 1.0.

**Table 6:** Equation 3 for stocks

	Equity class				
	Market	Health	Health services	Medical equipment	Drugs
α1	[−1.71,−1.36]	[−1.84,−1.22]	[−0.68,1.16]	[−1.87,−1.03]	[−2.39,−1.52]

This table shows the results for applying Equation 3 to the average rates and errors shown in Tables 3 and 4 using the returns to equities. The range of smallest to largest values of α_1_ are shown in the brackets. None of the results are statistically significant at the 10% level.

**Table 7:** Equation 3 for bonds

			Bond class
	10 Year Government Bonds	AAA Corporate Bonds	Risk-Free Rate
α1	[−1.18*,−0.91]	[−0.95**, −0.75*]	[−0.88***, −0.45**]

This table shows the results for applying Equation 3 to the average rates and errors shown in Tables 3 and 4 using the returns to bonds. The range of smallest to largest values of α_1_ are shown in the brackets. The asterisks denote levels of significance. One asterisk (*) denotes significance at the 10% level, i.e. p<0.10, two asterisks (**) denotes significance at the 5% level i.e. p<0.05, and three asterisks (***) denotes significance at the 1% level i.e. p<0.01.
